# Validation of secondary triage algorithms for mass casualty incidents: A simulation-based study—English version

**DOI:** 10.1007/s00101-023-01292-2

**Published:** 2023-10-12

**Authors:** Axel R. Heller, Tobias Neidel, Patrick J. Klotz, André Solarek, Barbara Kowalzik, Kathleen Juncken, Christan Kleber

**Affiliations:** 1 Department of Anesthesiology and Operative Intensive Care Medicine, Faculty of Medicine, University of Augsburg, Stenglinstraße 2, 86156 Augsburg, Germany; 2 Interdisciplinary Emergency Department, Medical Faculty, University Medical Center Schleswig-Holstein Campus Kiel, Kiel, Germany; 3 Department of Disaster preparedness and Emergency Planning, Charité, Berlin, Germany; 4 Division III.3 Protection of Health, German Federal Office for Civil Protection and Disaster Assistance, Bonn, Germany; 5 Medical Directorate, Dresden Municipal Hospital, Dresden, Germany; 6 Clinic and Polyclinic for Orthopaedics, Trauma Surgery and Plastic Surgery, University Hospital Leipzig AöR, Leipzig, Germany

**Keywords:** Katastrophenmedizin, Notfallvorsorge, Innerklinische Sichtung, Krankenhaus Alarm- und Einsatzplan, Notaufnahme, Disaster management, Emergency preparedness, Secondary triage, Hospital alarm and operation plan, Emergency department

## Abstract

**Background:**

In the event of a mass casualty incident (MCI), the situation-related shortage of medical resources does not end when the patients are transported from the scene of the incident. Consequently, an initial triage is required in the receiving hospitals. In the first step, the aim of this study was to create a reference patient vignette set with defined triage categories. This allowed a computer-aided evaluation of the diagnostic quality of triage algorithms for MCI situations in the second step.

**Methods:**

A total of 250 case vignettes validated in practice were entered into a multistage evaluation process by initially 6 and later 36 triage experts. This algorithm—independent expert evaluation of all vignettes—served as the gold standard for analyzing the diagnostic quality of the following triage algorithms: Manchester triage system (MTS module MCI), emergency severity index (ESI), Berlin triage algorithm (BER), the prehospital algorithms PRIOR and mSTaRT, and two project algorithms from a cooperation between the Federal Office of Civil Protection and Disaster Assistance (BBK) and the Hashemite Kingdom of Jordan—intrahospital Jordanian-German project algorithm (JorD) and prehospital triage algorithm (PETRA). Each patient vignette underwent computerized triage through all specified algorithms to obtain comparative test quality outcomes.

**Results:**

Of the original 250 vignettes, a triage reference database of 210 patient vignettes was validated independently of the algorithms. These formed the gold standard for comparison of the triage algorithms analyzed. Sensitivities for intrahospital detection of patients in triage category T1 ranged from 1.0 (BER, JorD, PRIOR) to 0.57 (MCI module MTS). Specificities ranged from 0.99 (MTS and PETRA) to 0.67 (PRIOR). Considering Youden’s index, BER (0.89) and JorD (0.88) had the best overall performance for detecting patients in triage category T1. Overtriage was most likely with PRIOR, and undertriage with the MCI module of MTS. Up to a decision for category T1, the algorithms require the following numbers of steps given as the median and interquartile range (IQR): ESI 1 (1–2), JorD 1 (1–4), PRIOR 3 (2–4), BER 3 (2–6), mSTaRT 3 (3–5), MTS 4 (4–5) and PETRA 6 (6–8). For the T2 and T3 categories the number of steps until a decision and the test quality of the algorithms are positively interrelated.

**Conclusion:**

In the present study, transferability of preclinical algorithm-based primary triage results to clinical algorithm-based secondary triage results was demonstrated. The highest diagnostic quality for secondary triage was provided by the Berlin triage algorithm, followed by the Jordanian-German project algorithm for hospitals, which, however, also require the most algorithm steps until a decision.

**Supplementary Information:**

The online version of this paper (10.1007/s00101-023-01292-2) contains the collection of additional figures S1 and S2 mentioned in the text and tables S1-S8.

## Abstract (de)

**Hintergrund:**

Die situationsbedingte Verknappung medizinischer Ressourcen endet bei einem Massenanfall von Verletzen (MANV) lageabhängig nicht mit dem Abtransport der Patienten von der Einsatzstelle. Folglich ist in den aufnehmenden Kliniken eine Eingangssichtung erforderlich. Ziel dieser Studie war es im ersten Schritt einen Referenz‐Patientenvignettensatz mit definierten Sichtungskategorien zu erstellen. Dies erlaubte im zweiten Schritt, die rechnergestützte Evaluation der diagnostischen Güte klinischer Sichtungsalgorithmen für MANV-Lagen.

**Methodik:**

In einen mehrstufigen Bewertungsprozess durch zunächst sechs, später 36 Sichtungsexperten gingen 250 in der Übungspraxis validierte Fallvignetten ein. Diese Algorithmen – unabhängige Expertenbewertung aller Vignetten – dienten als Goldstandard für die Analyse der diagnostischen Güte der folgenden innerklinischen Algorithmen: Manchester Triage System (MTS Modul MANV), Emergency severity Index (ESI), Berliner Sichtungsalgorithmus (BER), die prähospitalen Algorithmen PRIOR und mSTaRT, sowie zwei Projektalgorithmen aus einer Kooperation des Bundesamts für Bevölkerungsschutz und Katastrophenhilfe (BBK) mit dem Haschemitischen Königreich Jordanien – innerklinischer jordanisch-deutscher Projektalgorithmus (JorD) und prähospitaler Sichtungsalgorithmus (PETRA). Jede Patientenvignette durchlief computergestützt eine Sichtung durch alle angegeben Algorithmen, um vergleichend die Testgüte für alle Verfahren zu erheben.

**Ergebnisse:**

Von den ursprünglich 250 Vignetten konnte eine Sichtungsreferenzdatenbank mit 210 Patientenvignetten algorithmenunabhängig validiert werden. Diese bildeten den Goldstandard für den Vergleich der analysierten Sichtungsalgorithmen. Die Sensitivitäten für die innerklinische Detektion von Patienten der Sichtungskategorie I lagen zwischen 1,0 (BER, JorD, PRIOR) und 0,57 (MANV-Modul MTS). Die Spezifitäten lagen zwischen 0,99 (MTS und PETRA) und 0,67 (PRIOR). Gemessen am Youden-Index ergab sich bei BER (0,89) und JorD (0,88) die beste Gesamtperformance für die Detektion von Patienten der Sichtungskategorie I. Eine Übertriage ist am ehesten bei PRIOR, eine Untertriage beim MANV-Modul von MTS zu erwarten. Bis zum Entscheid SK I benötigen die Algorithmen folgende Schrittanzahlen (Median [IQR]): ESI 1 [1–2]; JorD 1 [1–4]; PRIOR 3 [2–4]; BER 3 [2–6]; mSTaRT 3 [3–5]; MTS 4 [4–5]; PETRA 6 [6–8]. Für die SK II und III besteht ein positiver Zusammenhang zwischen der Schrittanzahl bis zum Entscheid und der Testgüte.

**Schlussfolgerung:**

In der vorliegenden Studie konnte eine Übertragbarkeit prähospitaler algorithmenbasierter Vorsichtungsergebnisse auf die Ergebnisse klinischer Algorithmen gezeigt werden. Die höchste diagnostische Güte für die innerklinischen Sichtung lieferten BER und JorD, die allerdings auch die meisten Algorithmusschritte bis zum Entscheid benötigen.

## Introduction

The simultaneous spontaneous occurrence of a larger number of injured or ill patients regularly challenges both preclinical and clinical structures in emergency medicine [1–3]. Mass casualty incidents (MCI) are characterized, at least initially, by a significant lack of resources [4, 5]. At this point in the course of the task, the goal must be to use the available but sparse resources so efficiently that the survival of as many patients as possible can still be ensured with the best possible quality of life [5–7]. This is accomplished by early graded identification of patients according to their immediate treatment needs, with appropriate classification into one of four triage categories (T1–T4 or red/yellow/green/blue) and their labelling [7–9].

The primary and secondary triage algorithms presented here can be found in German legal terminology as ex ante triage: it is applied “… if the number of people to be treated exceeds the available resources, so that although all patients may have an alternative chance of successful treatment, this cannot be done at the same time” [10]. German case law regards this scenario for local actors as a collision of several equivalent obligations to act to save lives. “… According to the legal concept of the justifying conflict of duties, which is not regulated by law, but recognized as customary law, consequently the one who saves only as many people as possible according to the resource situation, does not act unlawfully” [10, 11].

In order to be able to implement this prioritization of treatment uniformly and precisely within a short time, different primary triage algorithms exist [12, 13]. These have increasingly been evaluated in studies in recent years [6, 13–17]. The 7th German Triage Consensus Conference held in 2017 at the German Federal Office for Civil Protection and Disaster Assistance (BBK) also drew up a requirement profile for primary triage algorithms considering previous evaluation data from our group [6, 18].

Depending on the situation, the shortage of resources does not end with the transport of patients from the scene but it continues to the recipient hospitals [1, 19] depending on the correct use of triage algorithms [5]. In this situation secondary triage of the patients must take place, firstly, to consider the dynamics of the patient’s condition over time [8, 9]. Secondly, due to the situation, it must also be expected that a primary triage cannot be carried out on site [20, 21].

The Manchester triage system (MTS) [22] and the emergency severity index (ESI) [23] are used as initial assessment tools in German emergency departments to prioritize patients’ first contact with physicians according to their current treatment urgency [3]; however, these two systems have not yet been evaluated for their suitability in MCI situations. Algorithms specifically tailored to secondary triage in MCI hardly exist. The utility of such algorithms has been demonstrated for prehospital emergency medicine [13]; however, overtriage or undertriage leads to patients not receiving the treatment resources that correspond to their actual treatment urgency [9, 24, 25]. Currently, the MCI module of the MTS [22] and the Berlin secondary triage algorithm [24] are the only procedures used in hospitals in Germany. The Berlin algorithm is the only one that has been validated internally and externally to date [24]. A systematic comparison of the different secondary triage algorithms based on standardized casualties is lacking.

The aim of this study was to create a consensus master data set of patient vignettes with defined triage categories, which also reflect the extended diagnostic capabilities of a hospital emergency department, in analogy to our own previous work focusing on the prehospital phase [6]. This makes it possible for the first time to evaluate existing secondary triage algorithms for patients in MCI situations. To ensure comparability with preclinical findings, the widely used primary triage algorithms PRIOR [26] and mSTaRT [16] were also included in the analysis. Likewise, two project algorithms from an international civil protection project of the BBK and the Hashemite Kingdom of Jordan are evaluated [27, 28].

## Methods

From the emergency drills of the Berlin and Dresden hospitals and emergency scenarios of the Berlin Fire Department, 250 case vignettes validated in practice without patient reference and with corresponding medical data were available [25]. The standardized vignettes were developed for the reproducible preparation of the mimes and as role scripts for the hospital emergency drills regularly held in Berlin from 2011–2015 [25]. The vignettes contained all relevant clinical information to simulate secondary triage in the emergency department and further care. In the case of corresponding injury patterns, the results of supplementary imaging (X-ray, sonography, computed tomography) were also available. The framework scenario was an out-of-hospital MCI, without resource limitation of the hospital itself [25]. In a two-stage Delphi procedure, the 250 case vignettes were first reviewed independently and blinded for plausibility and completeness by six members of the 8th German Triage Consensus Conference [8] and assigned to a corresponding triage category. In a second round of validation, inconsistently rated vignettes were discussed. Either consensus was reached or the case vignettes were removed from the vignette set. Likewise, duplicates were removed from further consideration. At the end of the preparatory process, 210 preconsented case vignettes remained for further refinement resulting in a reference data set in the subsequent broader validation round by a board of experts.

For broad consensus on the triage categories of the prepared patient vignette set and for further refinement to become the reference data set, the 210 remaining case vignettes were submitted to a group of 36 emergency physicians experienced in triage, analogous to prehospital advance work [6]. The experts were personally invited by the BBK or in coordination with the BBK. The evaluation consensus with respect to the appropriate triage category was password-protected on the SoSci Survey online survey platform [29]. A total of five clear evaluation examples were given to the experts per triage category for orientation. They were explicitly asked not to use algorithms, but to decide according to their clinical experience.

Thus, the formation of the reference categories is based on 7560 triage processes. According to the 8th German Triage Consensus Conference series held in 2019/2020 [8] only the triage categories T1 (red), T2 (yellow) and T3 (green) could be assigned for the entrance triage in the hospital. In accordance with the consensus of the professional societies involved, the triage category T4 (blue) was not available for the initial assessment in the hospital. The T4 (blue) category may be assigned as part of a re-evaluation in the T1 (red) treatment areas if there is an effective lack of resources. [8]. As an individual reference of the triage category for each vignette, the median of the triage categories determined by the 36 experts was used for the further comparison of the algorithms.

For the present study, the secondary triage algorithms Manchester triage system (MTS, module MCI [22]), emergency severity index (ESI [23, 30]), the Berlin secondary triage algorithm (BER [24]), the primary triage algorithms primary ranking for initial orientation in rescue (PRIOR [26]) and the modified simple triage and rapid treatment (mSTaRT [31]) as well as two algorithms from an international civil defense project of the BBK with the Hashemite Kingdom of Jordan, “JorD” (intrahospital, supplemental digital content, figure S1 [27]) and PETRA (prehospital, supplemental digital content, figure S2 [28]) were used. The aim of this international cooperation was to strengthen Jordanian civil emergency response by providing equipment assistance, training and conceptual advice. In the population health protection sub-project, prehospital and clinical aspects were considered and trained.

The primary triage algorithm PETRA was developed by Jordanian paramedics of the Jordan Civil Protection Department and German experts in 2018 at the BBK, later coordinated with the Jordanian Civil Defense Directorate and has since been trained nationwide. Jordanian and German doctors worked together for the development of the secondary triage algorithm “JorD”. In the workshop held in 2018 at the BBK criteria were developed, which were later combined into an algorithm and made available to the Jordanian Ministry of Health for further use and adaptation. As the prehospital algorithms PRIOR and mSTaRT had already been evaluated in preliminary work [6] and are widely used in Germany, these were included for external validation of the new reference dataset.

All algorithms were translated into Microsoft Excel syntax (Microsoft, Munich, Germany). For each patient case in the Excel database a triage category was automatically calculated according to the respective algorithms (supplemental digital content, tables S1–S7). In addition, the Excel syntax also indicates after how many steps the respective algorithm has been exited. For this purpose, the database had to be converted to an approximate binary format in that the columns contained the result of the respective query of the algorithms. In order to limit the complexity of the database, queries of the algorithms that were similar in meaning were combined (e.g. unstable pelvis and pelvic fracture to the common column pelvic fracture, respiratory rate > 29/min and respiratory rate > 30/min to the common column respiratory rate > 29/min, FAST (Focused Assessment with Sonography for Trauma) positive and FAST negative to the common column FAST). An overview of the adjustments and the queries of the algorithms can be found in the supplemental digital content. The result of the queries of the algorithms was coded as follows:

- “0” → query must be answered with “no”,
- “1” → query must be answered with “yes”,
- “*n*” → result of the query cannot be derived from the content of the existing dataset.

When querying the assumed number of resources required in the ESI algorithm, the ESI algorithm coded according to the ESI specifications “0 is no resources”, “1 is 1 resource” and “2 is many resources” [23]. The ESI levels 1 and 2 were assigned to the T1 (red) and T2 (yellow) triage categories, respectively and ESI levels 3–5 were grouped together into T3 (green).

After appropriate preparation of the database, the triage categories and the number of algorithm steps until a decision was made were calculated automatically for each patient vignette according to the respective algorithms. Subsequently, these triage categories generated by the algorithms were evaluated in comparison to the reference triage category with respect to their diagnostic quality analogous to our previous work in the prehospital setting [6]. The statistical evaluation was carried out with Microsoft Excel. Sensitivity, specificity, negative predictive value (NPV), positive predictive value (PPV) and Youden’ s index were determined for triage categories T1–T3. Youden’ s index [32] summarizes the sensitivity and specificity equally (Youden index = sensitivity + specificity − 1). In addition, the algorithms were also evaluated in terms of overtriage and undertriage. It should be noted that a patient vignette with T1 (red), in addition to its correct classification, can only be undertriaged (Table 1). For T2 (yellow), both other outcomes are possible in addition to the correct classification (Table 2). In the T3 category (green) it can only be correctly triaged or overtriaged (Table 3).

**Table 1:** Test quality of the examined triage algorithms for the detection of a condition according to triage category T1 (*red*). The higher the sensitivity, the lower the undertriage and the higher the specificity, the lower the overtriage

Triage category T1 (color: red)		BER	ESI	MTS	JorD	PETRA	PRIOR	mSTaRT
*Sensitivity*		1.00	0.80	0.57	1.00	0.73	1.00	0.92
*Specificity*		0.89	0.89	0.99	0.88	0.99	0.67	0.92
*PPV*		0.73	0.70	0.97	0.71	0.95	0.48	0.78
*NPV*		1.00	0.94	0.88	1.00	0.92	1.00	0.97
*Youden index*		0.89	0.69	0.57	0.88	0.72	0.67	0.84
*Undertriage*		0.0%	20.4%	42.9%	0.0%	26.5%	0.0%	8.2%
*Algorithm steps*	Correct	3 [2–3]	1 [1–1]	4 [4–4]	1 [1–3]	6 [6–6]	3 [2–3]	3 [3–4]
	Undertriage	None	2 [2–2]	5 [1–5]	None	1 [1–8]	None	1 [1–3.5]
	Overall	3 [2–6]	1 [1–2]	4 [4–5]	1 [1–4]	6 [6–8]	3 [2–4]	3 [3–5]

*PPV* positive predictive value, *NPV* negative predictive value. The Youden index [32] combines sensitivity and specificity in one value and increases with the discriminatory power of the algorithms. *BER* Berlin secondary triage algorithm [24], *JorD* Jordanian-German project hospital algorithm [27], *MTS* Manchester triage system MCI module [22], *ESI* emergency severity index [23]. Primary triage algorithms: *PETRA* prehospital emergency triage rapid algorithm [28], *PRIOR* primary ranking for initial orientation in emergency services [26], *mSTaRT* modified simple triage and rapid treatment [16]. Algorithm steps as medians [*IQR*], significance levels in the electronic supplementary material

**Table 2:** Test quality of the examined triage algorithms for the detection of a condition according to triage category T2 (*yellow*). The higher the sensitivity, the lower the undertriage and the higher the specificity, the lower the overtriage

Triage category T2 (color: yellow)		BER	ESI	MTS	JorD	PETRA	PRIOR	mSTaRT
*Sensitivity*		0.38	0.22	0.16	0.11	0.27	0.02	0.13
*Specificity*		0.90	0.78	0.73	0.99	0.75	0.99	0.81
*PPV*		0.52	0.21	0.14	0.83	0.22	0.33	0.16
*NPV*		0.84	0.79	0.76	0.80	0.79	0.79	0.77
*Youden index*		0.28	0.00	−0.11	0.11	0.01	0.01	−0.06
*Overtriage*		35.6%	33.3%	2.2%	40.0%	4.4%	51.1%	24.4%
*Undertriage*		26.7%	44.4%	82.2%	48.9%	68.9%	46.7%	62.2%
*Algorithm steps*	Correct	10 [9–14]	2 [2–2]	5 [5–5]	9 [9–9]	8 [8–8]	7 [7-7]	6 [6–6]
	Overtriage	3 [2–3]	1 [1–1]	4 [4–4]	4 [2–4]	6 [6–6]	5 [3–5]	5 [3–5]
	Undertriage	17 [17–17]	4 [4–4.5]	1 [1–1]	11 [11–11]	1 [1–1]	8 [8–8]	1 [1–1]
	Overall	9 [3–17]	2 [1–5]	1 [1–5]	9 [4–11]	1 [1–8]	6 [5–8]	1 [1–6]

Positive predictive value (*PPV*), negative predictive value (*NPV*). The Youden index [32] combines sensitivity and specificity in one value and increases with the discriminatory power of the algorithms. *BER* Berlin secondary triage Algorithm [24], *JorD* Jordanian-German Project Hospital Algorithm [27], *MTS* Manchester Triage System MCI Module [22], *ESI* Emergency Severity Index [23]. Primary triage algorithms: *PETRA* Prehospital Emergency Triage Rapid Algorithm [28], *PRIOR* Primary Ranking for Initial Orientation in Emergency Services [26], *mSTaRT* Modified Simple Triage and Rapid Treatment [16]

**Table 3:** Test quality of the examined triage algorithms for the detection of a condition according to triage category T3 (*green*). The higher the sensitivity, the lower the undertriage and the higher the specificity, the lower the overtriage

Triage category T3 (color: green)		BER	ESI	MTS	JorD	PETRA	PRIOR	mSTaRT
*Sensitivity*		0.84	0.74	0.72	0.97	0.68	0.72	0.72
*Specificity*		0.87	0.78	0.51	0.77	0.59	0.78	0.67
*PPV*		0.89	0.80	0.65	0.84	0.67	0.80	0.73
*NPV*		0.82	0.71	0.60	0.96	0.60	0.70	0.66
*Youden index*		0.72	0.52	0.23	0.74	0.27	0.50	0.39
*Overtriage*		15.5%	25.9%	27.6%	2.6%	31.9%	27.6%	28.4%
*Algorithm steps*	Correct	17 [17–17]	5 [5–5]	1 [1–1]	11 [11–11]	1 [1–1]	8 [8–8]	1 [1–1]
	Overtriage	8 [8–10]	2 [2–2]	5 [5–5]	4 [2–9]	8 [8–8]	6 [6–6]	6 [6–6]
	Overall	17 [17–17]	5 [2–6]	1 [1–5]	11 [11–11]	1 [1–8]	8 [6–8]	1 [1–6]

Positive predictive value (*PPV*), negative predictive value (*NPV*). The Youden index [32] combines sensitivity and specificity in one value and increases with the discriminatory power of the algorithms. *BER* Berlin secondary triage Algorithm [24], *JorD* Jordanian-German Project Hospital Algorithm [27], *MTS* Manchester Triage System MCI Module [22], *ESI* Emergency Severity Index [23]. Primary triage algorithms: *PETRA* Prehospital Emergency Triage Rapid Algorithm [28], *PRIOR* Primary Ranking for Initial Orientation in Emergency Services [26], *mSTaRT* Modified Simple Triage and Rapid Treatment [16]

The inferential statistical evaluation of the algorithm steps was performed with SPSS version 24 (IBM, Ehningen, Germany). In the absence of homogeneity of variances, medians with interquartile range (IQR) are given. For statistical comparison of the number of steps to decision by the algorithms, univariate analysis of variance with Dunnett T‑3 post hoc test for multiple testing without homogeneity of variances was used. Statistical significance was accepted at *p* < 0.05.

## Results

After initial removal of duplicates or vignettes for which no agreement in the evaluation could be found in the preparatory Delphi process, 210 patient vignettes were available for the construction of the reference database. The response rate of the evaluation was 100% due to the individual contractual agreement of the BBK with all experts involved. All triage procedures by the experts could be used for evaluation. Thus, 7560 triage processes of 36 experts were available for the formation of the patient vignette reference database. The median triage results for each of the 210 patient vignettes resulting from this reference formation provided the gold standard for comparing the 7 triage algorithms with each other.

**Fig. 1:**
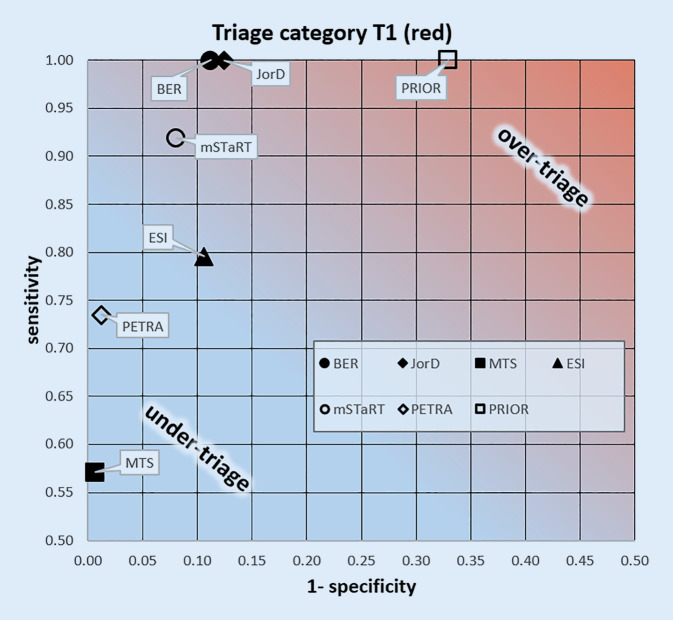
Test quality of triage algorithms for the identification of severely injured patients of triage category T1 (*red*). Filled symbols secondary triage algorithms. *BER* Berlin secondary triage algorithm [24]; *JorD* Jordanian-German Project Hospital algorithm [27]; *MTS* Manchester triage system-MCI module [22]; *ESI* emergency severity index [23]. Empty symbols Primary triage algorithms: *PETRA* prehospital emergency triage rapid algorithm [28]; *PRIOR* primary ranking for initial orientation in emergency services [26]; *mSTaRT* modified simple triage and rapid treatment [16], *sensitivity *proportion of correct inclusions, *specificity* proportion of correct exclusions

Figure 1 shows the analysis of the algorithms with respect to triage category T1 (red) in a receiver-operating characteristic. Detailed results are shown in Table 1. The calculated sensitivities for the detection of triage category T1 patients ranged from 1.0 (Berlin algorithm, JorD and PRIOR) to 0.57 (MANV module MTS). Specificities ranged from 0.99 (MTS and PETRA) to 0.67 (PRIOR). The highest sensitivity for detection of a triage category T1 (red) was achieved by the Berlin triage algorithm, JorD and PRIOR. The algorithms MTS and PETRA showed the highest specificity. Considering the Youden index, the Berlin triage algorithm showed the best overall performance (0.89), immediately followed by the intrahospital Jordan-German Project algorithm (JorD) with 0.88. Of the algorithms evaluated here, PRIOR is most likely to overtriage and the MANV module of MTS is most likely to undertriage.

Overall, the accuracy of the algorithms for the detection of patients in triage category T2 (yellow) and T3 (green) is significantly worse than for those in triage category T1 (red). For triage category T2 (yellow) (Table 2) the calculated sensitivities ranged from 0.38 (BER) to 0.02 (PRIOR). Specificities ranged from 0.99 (JorD and PRIOR) to 0.73 (MTS). When considering the Youden index, the Berlin triage algorithm also showed the best overall performance (0.28).

For the detection of patients in triage category T3 (green) (Table 3), the calculated sensitivities ranged from 0.97 (JOR) to 0.68 (PETRA). Specificities ranged from 0.87 (BER) to 0.51 (MTS). When considering the Youden index, JorD (0.74) and BER (0.72) showed the best overall performance for the detection of patients in triage category T3 (green).

Across all triage categories, the following numbers of steps (median [IQR]) in descending order were necessary until a decision was made by the different algorithms: BER 17 [3–17]; JorD 11 [4–11]; PRIOR 7.5 [4–8]; ESI 4 [1–5]; PETRA 1 [1–8] and 1 [1–4] steps, both with mSTaRT and the MANV module of MTS. Table 1, 2 and 3 give the respective number of steps separately by triage category and achieved correctness. The differences had a *p*-value of < 0.001 in the ANOVA. The results of the individual comparisons are given in the electronic supplementary material (Table S8).

## Discussion

In recent years more studies have been conducted to evaluate primary triage algorithms for use on scene [6, 13–17]. In contrast, there is a lack of studies on secondary triage algorithms designed for use during admission to the hospital in an MCI scenario. In particular, reliable recommendations are not available for the current German guideline process in clinical disaster medicine [33]. For this reason, the aim of the present study was to evaluate and compare existing and newly developed secondary triage algorithms. One of the main results of this study is the establishment of a master dataset of 210 casualty vignettes, which were validated by a total of 36 national experts. This master dataset offers the possibility to validate new triage algorithms and improve existing algorithms in the future. To establish comparability with studies on primary triage algorithms, PRIOR [26], mSTaRT [16] and PETRA (supplemental digital content, figure S2) were also included in the analyses.

Triage algorithms aim to classify MCI patients into a triage category as precisely as possible according to their injury pattern. Here, the triage category T1 (red) is of utmost relevance, as there is an immediate and acute danger to the lives of these patients [8, 9]. Therefore, it is of paramount importance that triage concepts reliably recognize these patients in particular.

In previous studies [6, 14, 15] we were able to show that especially the algorithms of the START family meet this requirement on scene, whereas PRIOR [26] significantly overtriages and the field triage score [34] significantly undertriages. In the present study, the sensitivity for the patients in triage category T1 (red) was more than 0.8 across most algorithms (Table 1). The MCI module of Manchester triage (MTS) stands out as a negative outlier with a sensitivity of only 0.57. Likewise, a high specificity of substantially more than 0.8 was shown for the detection of triage category T1 (red) for most algorithms (Table 1). Similar to the prehospital evaluation [6], the specificity of the PRIOR algorithm with 0.67 was shown to be the taillight.

Thus, it can be stated that the investigated algorithms meet the requirements of a precise recognition of patients of T1 to varying degrees. This is also consistent with previous studies [6, 16, 35]; however, it is an inherent problem of all diagnostic tests that high sensitivity, i.e., the detection of all life-threatening injuries, can only be achieved at the expense of specificity and makes overtriage more likely (Fig. 1). In order to enable a balanced consideration of sensitivity and specificity (here danger of undertriage), the Youden index [32] is given for all investigated algorithms, which combines both sensitivity and specificity into one reading indicating a higher ability to discriminate with increasing values.

With respect to the comparability of primary to secondary triage algorithms, there is a largely good agreement (Fig. 1). This is particularly noteworthy from a process quality point of view as it facilitates the translation of information from the scene to the hospital [5]. In particular, the prehospital triage results correspond to the ESI levels, based on the chosen assignment. This enables the respective teams (prehospital vs. clinical) to apply their usual algorithms without causing breaks in the classification into the triage categories or the ESI levels. The calculations have yielded remarkably consistent results, especially for the transfer of triage category T1 to ESI level 1. In addition, the data show that T3 patients must always be grouped into ESI levels 3–5. A more precise subdivision of ESI levels 3–5 can then be done in the emergency department, e.g., in the entrance triages.

The highest test quality for the detection of the T1 (red) patients was, however, provided by the Berlin triage algorithm. With a sensitivity of 1.0 and a specificity of 0.89, it is the most balanced algorithm within this analysis in terms of overtriage and undertriage; but closely followed by the Jordanian-German project algorithm (JorD) (figure S1 [27]) and by mSTaRT [16]. The MTS and ESI algorithms used in emergency departments showed worse results in comparison, which is why the authors recommend providing a special clinical triage algorithm for MCI.

The Akaike information criterion [36] requires that a model with the same quality is to be preferred which has a lower complexity. Accordingly, the number of parameters or query items is to be taken into account in a “punitive” way. Transferred to triage algorithms, a simpler algorithm is faster in execution [6, 37] and also easier to train [18, 38]. In addition, simple algorithms can also be executed unproblematically in the form of checklists that can be processed more quickly [39, 40], but possibly with the restriction of lower precision as will be explained later.

In the present study the duration of the algorithm runs could not be determined comparatively on the basis of the computer-aided simulation as it would have been possible with human subjects. Alternatively, only the number of steps passed until the result could be used [6]. From an evaluation study of the PRIOR algorithm [37] it is known that the triage of T3 (green) patients took the longest (42 s) compared to both the other triage categories and the mSTaRT algorithm. The triage process was reported to be 27/28/42 s with PRIOR for T1/T2/T3 and 35/20/10 s for mSTaRT, respectively. If time approaches are compared, the distribution of triage categories in the cohort under consideration must be considered. With a patient distribution of T1/T2/T3/dead of 15%/20%/60%/5% in 100 patients triaged with PRIOR, 42 min are required only for T3 patients. In comparison, the time required by mSTaRT for this category is 10 min. If the patient distribution shifts even further in favor of T3, as in the overall distribution found by Brüne [2] for MCI in Germany (T1/T2/T3 of 7%/19%/74%), then 81% of the triage time with the PRIOR method is used for only lightly injured patients. Variations in item sequence within an algorithm [15] or their premature termination can positively or negatively affect both diagnostic accuracy and time to decision, depending on the algorithm.

Across the triage categories, both BER with 17 [3–17]; and JorD with 11 [4–11] require significantly more steps than all other algorithms; however, this isolated and cross-category view is not suitable for assessing the degree to which triage algorithms fulfil their task. According to the 7th German Triage Consensus Conference [18], algorithms should have the following characteristics in descending importance:

- Rapidly identify T1 (red) patients.
- Reliable identification (avoid overtriage and undertriage)
- Low time requirement
- Easy to use
- Easy to learn

**Fig. 2:**
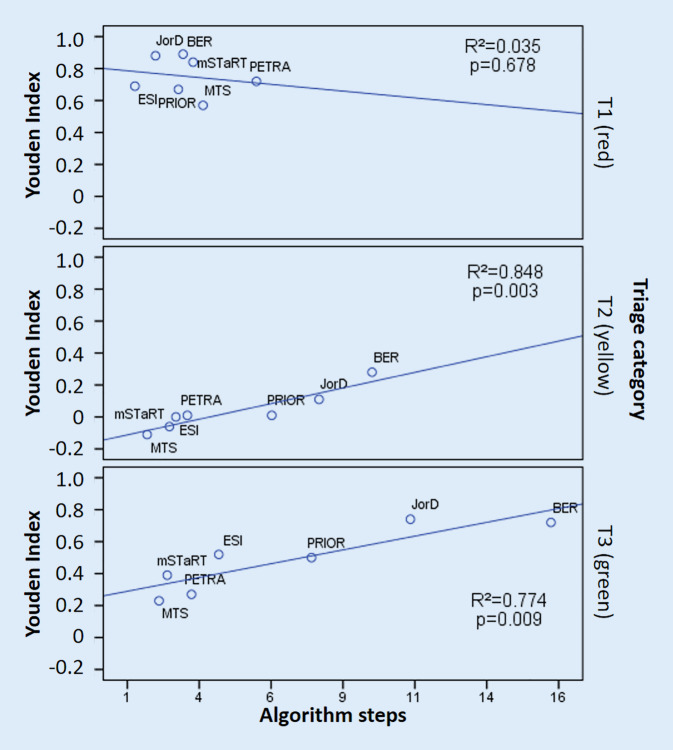
Test quality (Youden index) of the triage algorithms in relation to the average algorithm steps required to decision by triage category. *BER* Berlin Hospital Algorithm [24], *JorD* Jordanian-German Project Hospital Algorithm [27], *MTS* Manchester Triage System-MCI Module [22], *ESI* Emergency Severity Index [23], *PETRA* Prehospital Emergency Triage Rapid Algorithm [28], *PRIOR* Primary Ranking for Initial Orientation in Emergency Services [26], *mSTaRT* Modified Simple Triage and Rapid Treatment [16]

Therefore, a comparative assessment of the time required or that of a surrogate parameter such as the number of algorithm steps by triage category is relevant. ESI (1 [1–1]) and JorD (1 [1–3]) require the fewest steps to correctly identify T1 (red) patients, yet ESI triages 20.4% of actual T1 (red) patients into T2 (yellow) (Table 1); however, according to Fig. 2 (top), no statistical association can be found between test quality and the algorithm steps at T1 (red). The problem of simpler triage algorithms is reflected particularly in the discrimination of T2 (yellow) patients, as misclassification in both directions is possible here [24]. This effect was demonstrated during the development and validation of the Berlin triage algorithm where the number of discriminants was increased from 5 yellow discriminants to 9 in favor of better discrimination [24]. Accordingly, BER requires the most steps to correctly identify T2 patients, with a total of 10 [9–14] (Table 3). This resulted in an improvement in accuracy but to the detriment of the number of discriminants. Higher accuracy therefore comes at the price of a more complex algorithm (T2 Youden index BER 0.28 vs. JorD 0.11 and Fig. 2 (middle)).

If the time required for the identification of light casualties (T3 green) is considered, it must be stated that the algorithms with the best discriminatory ability require the most steps and thus also need the longest time for a correct decision Fig. 2 (below): BER (17 [17–17]) vs. JorD (11 [11–11]) steps; however, this is due to prioritization of detection of T1 patients. In regularly performing ambulance services with transport prioritization during MCI, T1 patients will arrive first in hospitals. The exception to this is scenarios with a relevant number of self-referrals. This problem has already been discussed above for the PRIOR algorithm: If, in a random arrival scenario, it is clear that the greatest time expenditure is for the identification of the lightly injured, then it must be ensured that no severely injured patient in a queue has to wait for the correct identification of lightly injured patients. To solve this dilemma, the 8th German Triage Consensus Conference already advocated access coordination to the triage point. “Depending on the situation, medical or healthcare professionals may coordinate access to the hospital with the goal of identifying patients who are obviously in vital threat. This is intended to expedite these patients to the triage process. This approach does not replace clinical triage” [8].

Independently, it has been shown for the prehospital PRIOR algorithm that simply moving the query item “ability to walk” from the end to the beginning of the algorithm not only significantly improves its discriminatory ability [15]. It further results that the total number of algorithm steps to be processed for a patient cohort decreases as a result of such a change. Accordingly, it should be examined whether the algorithms investigated here could be further improved by a similar rearrangement of the query items.

Another aspect is the time required for administrative tasks in the hospitals. From the authors’ experience, this takes the most time at the triage site, despite prepared disaster files, so that the time required by the triage algorithm itself tends to recede into the background. Taking these discussions into account, it is ultimately up to the user to decide which strategy to pursue in the context of secondary triage. Here, above all, the time required by a more complex algorithm at the triage site must be weighed against the possibly poorer diagnostic quality of a simpler algorithm. In the case of a less pronounced lack of resources, the overtriage by a certain algorithm has less negative effects on the competition of the true T1 patients (true positives) for the medical resources with the incorrectly assigned T1 patients (false positives). From the algorithm training point of view of permanent knowledge availability and secure application, it is inadvisable to maintain algorithms that differ according to location and resources [8].

Another point of discussion is the use of a focused ultrasound examination (FAST) [41] in secondary triage algorithms. In principle, it seems to make sense to include resources available within the hospital that can contribute to a better discrimination of the patient’s condition in the triage process, which are not realistically available and usable on scene. For example, personnel trained and experienced in FAST can provide valuable information that can guide the next steps of treatment. At the same time, it should be noted that focusing FAST on those patients who have undergone a sensitive preselection process, such as triage based on clinical parameters, will more accurately target the specialist resource. If scenario-dependent category distributions of T1 (20%)/T2 (30%)/T3 (50%) are taken as a basis [2, 9, 18, 42], then more than 50% of the FAST examinations are dispensable. This offers the possibility of dealing more extensively with the patients of T1 in the treatment area “red” [3, 41]. The training concept of the Berlin secondary triage algorithm takes this into account: Only patients with blunt abdominal trauma without acute vital threat receive FAST sonography at the point of triage in order to be able to distinguish patients with free intra-abdominal fluid (T1) from patients without (T2). Such personnel are regularly available in emergency departments. In prehospital settings, however, training and use of FAST is less established [43].

The BBK co-financed the development of the underlying patient data set, which can be used in the future for national and international training and exercises in hospital disaster planning. The evaluation results of the triage algorithms developed for the international civil defense project of BBK with the Hashemite Kingdom of Jordan are a confirmation of the successful international project cooperation and increase the acceptance of the algorithms by decision makers in both partner countries.

A limiting factor in the present analysis is that the elaborated patient vignettes from the Berlin hospital emergency exercises [25] all represent fictitious case studies, in contrast to our previous prehospital study from real patient cases [6]. In addition, the design of the database itself is limiting. As the queries of the triage algorithms are very specific in many cases, a column with the respective information of the patient example did not exist 1 to 1 for each algorithm item and had to be recalculated in a complex way. In a subsequent study, therefore, greater care should be taken to ensure that all queries of the algorithms under investigation are directly reflected in the patient database.

## Conclusion

In the present study, transferability of prehospital algorithm-based primary triage results to clinical algorithm-based secondary triage results was demonstrated. The highest diagnostic quality for secondary triage was provided by the Berlin triage algorithm, followed by the Jordanian-German Project (JorD) algorithm which, however, also require the most algorithm steps to come to a decision. In a subsequent study, the results need to be validated on real patient datasets, e.g., from emergency departments. Further research is still needed for a possible improvement of the algorithms themselves.

### Supplementary Information

Additional figures and tables

## References

[1] Adams H, Paal P (2015). Patientenversorgung im Großschadens- und Katastrophenfall. Med Klin Intensivmed Notfmed.

[2] Brüne F (2013). Reale Verteilung von Sichtungskategorien bei MANV Einsätzen – Auswirkungen auf die Schutzziele.

[3] Heller AR, Juncken K (2020). Primärversorgung in der Zentralen Notaufnahme. Anasth Intensivmed.

[4] Heller AR (2006). Katastrophenmedizin: Wunsch und Wirklichkeit. Dtsch Arztebl Int.

[5] Heller AR, Juncken K, Knickmann AO, Piepho T (2020). Einsatzführung durch den LNA. Handbuch für den Organisatorischen Leiter und Leitenden Notarzt.

[6] Heller AR, Salvador N, Frank M, Schiffner J, Kipke R, Kleber C (2019). Diagnostic precision of triage algorithms for mass casualty incidents. English version. Anaesthesist.

[7] AWMF (2019). Anmeldung: S2k-Leitlinie Katastrophenmedizinische präklinische Behandlungsleitlinien. Registernummer 001-043.

[8] BBK Protokoll 8. Sichtungs-Konsensus-Konferenz2019. https://www.bbk.bund.de/SharedDocs/Downloads/DE/Gesundheit/Sichtung/protokoll-8sikokon-download.pdf?__blob=publicationFile&v=4. Accessed 12.5.2023

[9] Heller AR, Brüne F, Kowalzik B, Wurmb T (2018). Großschadenslagen: Neue Konzepte zur Sichtung. Dtsch Arztebl Int.

[10] Rönnau T, Wegner K (2020). Grundwissen – Strafrecht: Triage. Jus.

[11] Heller AR, Bartenschlager CC, Brunner JO, Marckmann G (2023) Das „Triagegesetz“ – Regelung mit fatalen Folgen. Anaesthesiologie 72. 10.1007/s00101-023-01286-0 10.1007/s00101-023-01286-0 PMC10215064 37233790

[12] Streckbein S, Kohlmann T, Luxen J, Birkholz T, Pruckner S (2016). Triage protocols for mass casualty incidents : An overview 30 years after START. Unfallchirurg.

[13] Bazyar J, Farrokhi M, Khankeh H (2019). Triage systems in mass casualty incidents and disasters: a review study with a worldwide approach. Open Access Maced J Med Sci.

[14] Neidel T, Salvador N, Heller AR (2017). Impact of systolic blood pressure limits on the diagnostic value of triage algorithms. Scand J Trauma Resusc Emerg Med.

[15] Neidel TH, Heller AR (2021). Einfluss der Reihenfolge von Items auf die diagnostische Qualität von Vorsichtungsalgorithmen hinsichtlich der Vergabe der Sichtungskategorie I. Notfall Rettungsmed.

[16] Paul AO, Kay MV, Huppertz T, Mair F, Dierking Y, Hornburger P (2009). Validierung der Vorsichtung nach dem mSTaRT-Algorithmus: Pilotstudie zur Entwicklung einer multizentrischen Evaluation. Unfallchirurg.

[17] Dittmar MS, Bigalke M, Brunner A, Hannewald W, Honig D, Honig M (2013). Ein regional angepasstes Vorgehen zur Vorsichtung und Sichtungskennzeichnung beim Massenanfall von Verletzten. Notarzt.

[18] BBK Protokoll 7. Sichtungs-Konsensus-Konferenz2017. https://www.bbk.bund.de/SharedDocs/Downloads/DE/Gesundheit/Sichtung/protokoll-7sikokon-download.pdf?__blob=publicationFile&v=7. Accessed 12.05.2023

[19] Pfenninger E, Königsdorfer M (2019). Exit wave plan for structured secondary patient distribution : Logistic concept for mass victims of terrorist attacks. Anaesthesist.

[20] Hossfeld B, Adams H, Bohnen R (2017). e. Zusammenarbeit von Rettungskräften und Sicherheitsbehörden bei bedrohlichen Lagen – Ergebnisse eines nationalen Konsensusgesprächs. Anasth Intensivmed.

[21] Sefrin P (2014). Vorsichtung notwendig – Bericht von der Nachfolge Sichtungskonferenz 2013. Notarzt.

[22] Kogej M, Kern M, Tralls P, Berger M, Gräff I (2021). Das Präsentationsdiagramm „Massenanfall“ des Manchester-Triage-Systems. Notfall Rettungsmed..

[23] Grossmann F, Delport K, Keller D (2009). Emergency severity index. Notfall Rettungsmed.

[24] Kleber C, Solarek A, Cwojdzinski D (2020). Der Berliner Krankenhaus-Sichtungsalgorithmus für den Massenanfall von Verletzten. Unfallchirurg.

[25] Kleber C, Cwojdzinski D, Strehl M, Poloczek S, Haas NP (2013). Results of in-hospital triage in 17 mass casualty trainings: underestimation of life-threatening injuries and need for re-triage. Am J Disaster Med.

[26] Bubser F, Callies A, Schreiber J, Grüneisen U (2014). PRIOR: Vorsichtungssystem für Rettungsassistenten und Notfallsanitäter. Rettungsdienst.

[27] Bundesamt für Bevölkerungsschutz und Katastrophenhilfe (2018) Entwicklung eines innerklinischen Sichtungsalgorithmus im Rahmen eines internationalen Zivilschutzprojektes des BBK mit dem Haschemitischen Königreich Jordanien. https://www.bbk.bund.de/DE/Themen/Internationale-Angelegenheiten/Internationale-Projektarbeit/Jordanien/jordanien_node.html. Accessed 12.05.2023

[28] Bundesamt für Bevölkerungsschutz und Katastrophenhilfe (2018) Prehospital Emergency Triage Rapid Algorithm (PETRA) – Entwicklung eines präklinischen Sichtungsalgorithmus – Internationales Zivilschutzprojekt des BBK mit dem Haschemitischen Königreich Jordanien. https://www.bbk.bund.de/DE/Themen/Internationale-Angelegenheiten/Internationale-Projektarbeit/Jordanien/jordanien_node.html. Accessed 12.05.2023

[29] Leiner DJ (2019). SoSci survey Onlinebefragungsinstrument (version 3.1. 06).

[30] Christ M, Grossmann F, Winter D, Bingisser R, Platz E (2010). Modern triage in the emergency department. Dtsch Arztebl Int.

[31] Hiereth K, Hornburger P, Eyer F, Gerstenhöfer S, Schmöller G, Prückner S (2013). mSTaRT Trauma & Intox. Notfall Rettungsmed.

[32] Youden WJ (1950). Index for rating diagnostic tests. Cancer.

[33] AWMF (2021). Ameldung: S2k-Leitlinie Behandlungsleitlinien und Behandlungsstrategien für den Einsatz in klinischen Krisen- und Katastrophenmedizin. Registernummer 187-048.

[34] Eastridge BJ, Butler F, Wade CE, Holcomb JB, Salinas J, Champion HR (2010). Field triage score (FTS) in battlefield casualties: validation of a novel triage technique in a combat environment. Am J Surg.

[35] Wolf P, Bigalke M, Graf BM, Birkholz T, Dittmar MS (2014). Evaluation of a novel algorithm for primary mass casualty triage by paramedics in a physician manned EMS system: a dummy based trial. Scand J Trauma Resusc Emerg Med.

[36] Akaike H (1998). Information theory and an extension of the maximum likelihood principle. Selected papers of Hirotugu Akaike.

[37] Zahn T, Schreiber J (2015). PRIOR Evaluierung – Design und Ergebnisse. Symposium 21112015: Medizinisches Management im MANV unter Verwendung der PRIOR-Vorsichtungssystems.

[38] BBK Protokoll 6. Sichtungs-Konsensus-Konferenz2015. https://www.bbk.bund.de/SharedDocs/Downloads/DE/Gesundheit/Sichtung/protokoll-6sikokon-download.pdf?__blob=publicationFile&v=8. Accessed 12.05.2023

[39] Offterdinger M, Ladehof K, Paul AO, Hansen M (2014). Eine einfache Checkliste als Hilfsmittel zur Vorsichtung mit dem mSTaRT-Algorithmus – Erste Erfahrungen aus der Simulation. Notfall Rettungsmed.

[40] Hügle C, Neidel T, Helmert J, Heller A (2018). Evaluationsstudie zur Nutzung von Checklisten vs. Flussdiagrammen bei der Vorsichtung, Projekt-Abschlussbericht. Dresden; 2018.

[41] Williams SR, Perera P, Gharahbaghian L (2014). The FAST and E-FAST in 2013: trauma ultrasonography: overview, practical techniques, controversies, and new frontiers. Crit Care Clin.

[42] Juncken K, Heller AR, Cwojdzinski D, Disch AC, Kleber C (2019). Distribution of triage categories in terrorist attacks with mass casualties : Analysis and evaluation of European results from 1985 to 2017. Unfallchirurg.

[43] Scharonow M, Weilbach C (2018). Prehospital point-of-care emergency ultrasound: a cohort study. Scand J Trauma Resusc Emerg Med.

