# Supplementary material for: Validation of secondary triage algorithms for mass casualty incidents: A simulation-based study—English version
Source: Anaesthesiologie. 2023 Oct 12;72(Suppl 1):1–9. doi: 10.1007/s00101-023-01292-2 (PMC10692258; doi:10.1007/s00101-023-01292-2)
Supplement: Supplementary file 1 — Additional figures and tables [file 101_2023_1292_MOESM1_ESM.docx]

**Supplemental material to the article** „**Validation of secondary triage algorithms for mass casualty incidents-**

**A simulation-based study**“ of Heller AR, Neidel T, Klotz PJ et al. (2023) in *Die Anaesthesiologie*.

The article and additional material are available at www.springermedizin.de. Please enter the title of the article in the search field.

**Figure S1: Jordanian-German secondary triage algorithm for hospitals (JorD) (**[**27**](#_ENREF_27)**) Triage categories T1-T4**


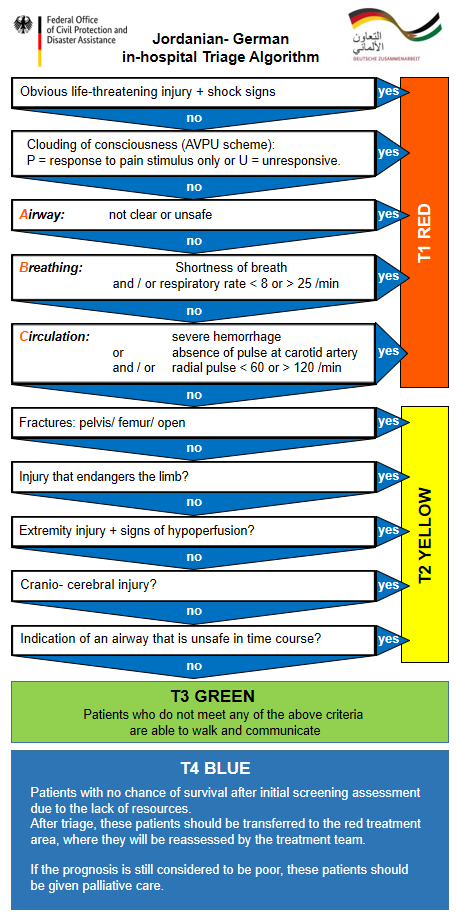


**Figure S2: Jordanian-German primary triage algorithm "Prehospital Emergency Triage Rapid Algorithm (PETRA) (**[***28***](#_ENREF_28)**) Triage categories T1-T4**


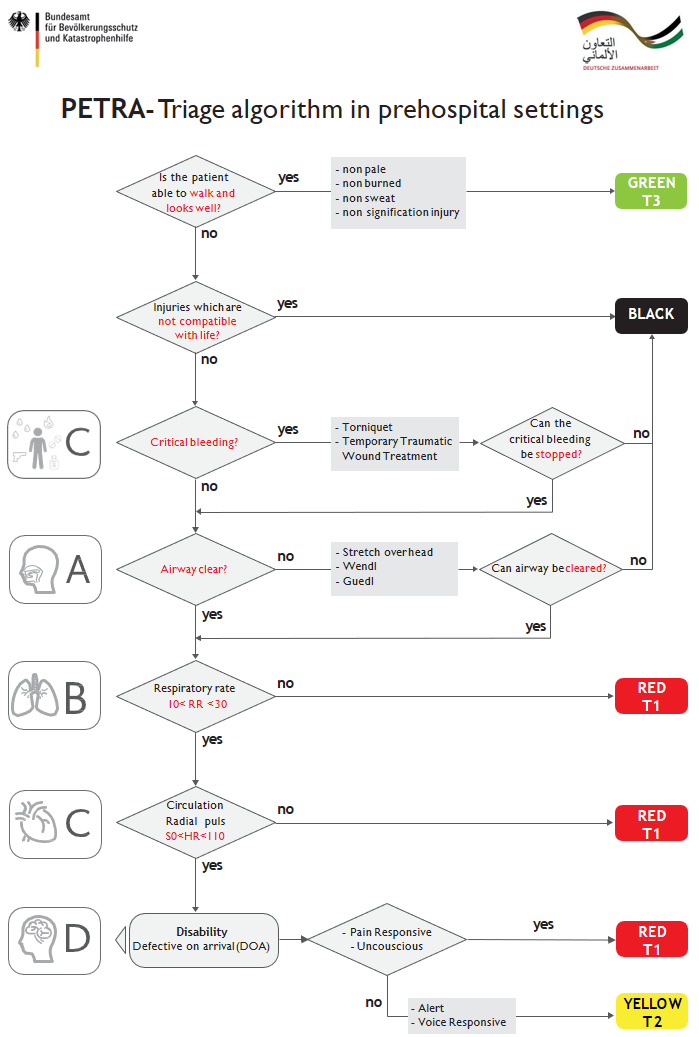


**Computer simulation of the algorithms**

The nested IF queries of the parameters from the corresponding parameter table for each algorithm determine the corresponding triage category and the number of algorithm steps performed on each patient. The IF function is commonly used to perform conditional tests on values and formulas, and returns a value that depends on whether a specified condition evaluates to TRUE or FALSE.

Syntax: IF(CHECK_TRUE; THEN_VALUE; ELSE_VALUE)

**Table S1: Parameter list for querying by the Excel syntax for the Berlin algorithm (BER) (**[***24***](#_ENREF_24)**)**

| Query | Column |
| --- | --- |
| Lack of spontaneous breathing | T |
| GCS<9 | AN |
| Respiratory insufficiency | W |
| Penetrating chest injury | AP |
| Shortness of breath | AA |
| Uncontrolled hemorrhage | AC |
| Mechanically unstable pelvis | BH |
| Abdominal trauma | AU (blunt) |
| Penetrating abdominal trauma | AQ |
| FAST positive | AD |
| Shock | AK |
| Recap time > 2 s or syst. Blood pressure <90 mmHg | AF or AE |
| Inhalation trauma | Z |
| Chest pain | AR |
| Shortness of breath | AA |
| Severe head injury | AM |
| Cross-section/paralysis | AL |
| Pelvic fracture | BH |
| Femoral fracture | AS |
| Open fracture | AT |
| Abdominal trauma blunt and FAST negative | AU and AD neg |
| Extensive soft tissue injury/ macro amputation | AV |
| Burns > 20% body surface are | AW |
| Electric accident | AX |
| Penetrating eye injury | AY |
| T3 (green) |  |

MS Excel syntax for determining the triage categories Berlin algorithm (BER) (21)
with variable reference to table 4:

=IF(T2=0;"SK1(1)';IF(AN2=1;"SK1(2)';IF(OR(W2=1;AP2=1;AA2=1);"SK1(3)';IF(AC2=1;"SK1(4)';IF(BH2=1;"SK1(5)';IF(OR(AU2=1;AQ2=1;AD2=1);"SK1(6)';IF(OR(AK2=1;AF2=1;AE2=1);"SK1(7)';IF(Z2=1;"SK2(8)';IF(OR(AR2=1;AA2=1);"SK2(9)';IF(AM2=1;"SK2(10)';IF(AL2=1;"SK2(11)';IF(OR(BH2=1;AS2=1;AT2=1);"SK2(12)';IF(AND(AU2=1;AD2=0);"SK2(13)';IF(AV2=1;"SK2(14)';IF(OR(AW2=1;AX2=1);"SK2(15)';IF((AY2=1);"SK2(16)";"SK3(17)"))

**Table S2: Parameter list for querying by the Excel syntax for the Emergency severity Index (ESI) (**[23](#_ENREF_23)**)**

| Query | Column |
| --- | --- |
| A: |  |
| Intubated | U |
| Apnea | T |
| No pulse | AG |
| Shortness of breath | AA |
| SpO_2_ <90 | W |
| Acute change in consciousness | AN |
| Unresponsive | AO |
|  |  |
| B |  |
| Thoracic pain | AR |
| Circulatory disorder | AU |
| Confusion | AN |
| Lethargy | AN |
| Disorientation | AN |
| Severe pain | AZ |
|  |  |
| C |  |
| Resources required | BJ |
| None -> 5 , one -> 4 , many -> D |  |
| D |  |
| Heart rate >100/ min | AH |
| Respiratory rate >20/ min | Y or AA? |
| SpO_2_ <92 | W or O |

MS Excel syntax for determining the triage categories for the Emergency severity Index (ESI) ([23](#_ENREF_23)) with variable reference to table 5:

=IF(OR(U2=1;T2=0;AG2=0;AA2=1;W2=1;AN2=1;AO2=0);"EL1";IF(OR(AR2=1;AU2=1;AN2=1;AZ2=1);"EL2";IF(AND(BJ2=2;(OR(AH2=1;Y2=1;W2=1)));"EL2";IF(AND(BJ2=2;AH2=0;Y2=0;W2=0);"EL3";IF(BJ2=1;"EL4";IF(BJ2=0;"EL5";"ERROR"))))))

Error output due to too little information of the AF in the database -> conversion query AF into query shortness of breath -> then result possible

=IF(OR(U2=1;T2=0;AG2=0;AA2=1;W2=1;AN2=1;AO2=0);"EL1";IF(OR(AR2=1;AU2=1;AN2=1;AZ2=1);"EL2";IF(AND(BJ2=2(OR(AH2=1;AA2=1;W2=1)));"EL2";IF(AND(BJ2=2;AH2=0;AA2=0;W2=0);"EL3";IF(BJ2=1"EL4";IF(BJ2=0;"EL5";"ERROR"))))))

**Table S3: Parameter list for querying by the Excel syntax for the MCI module of the Manchester Triage System (MTS) (**[**22**](#_ENREF_22)**)**

| Query | Column |
| --- | --- |
| Walkable | BA |
| Spontaneous breathing | T and U (not intubated) |
| Respiratory rate <10/min | X |
| Respiratory rate > 29/min | Y |
| Recap time > 2s | AF |
| Heart rate > 120/min | AH |
| T2 (yellow) |  |

MS Excel syntax for determining the triage categories for the MCI module of the Manchester Triage System (MTS) ([22](#_ENREF_22)) with variable reference to table 6:

=IF(BA2=1;"SK3(1)';IF(AND(T2=0;U2=0);"EX(2)';IF(OR(X2=1;Y2=1);"SK1(3)';IF(OR(AF2=1;AH2=1);"SK1(4)";"SK2(5)"))))

***Table S4: Parameter list for querying by the Excel syntax for the Jordanian-German project algorithm for hospitals ((JorD) SDC figure 3(***[**27**](#_ENREF_27)***))***

| Query | Column |
| --- | --- |
| Life-threatening injury + shock | AK and BG |
| Clouding of consciousness | AT |
| Airway not free or unsafe | T or V + not intubated |
| Shortness of breath | AA |
| Respiratory rate <8 or > 25/min | X and Y |
| Uncontrolled hemorrhage | AC |
| Heart rate <60 or >120/ min | AH and AI |
| Missing pulse | AG |
| Pelvic fracture | BH |
| Femoral fracture | AS |
| Open fracture | AT |
| Extremity at risk | BI |
| Extremity injury + signs of reduced perfusion | BI |
| Craniocerebral injury | AM |
| Unsafe airway | V |
| T3 (green) |  |

MS Excel syntax for determining the triage categories for ((JorD) SDC figure 3 (23)) with variable reference to table 7:

=IF(AND(AK2=1;BG2=1);"SK1(1)';IF(AN2=1;"SK1(2)';IF(OR(and(T2=0;U2=0);V2=1);"SK1(3)';IF(OR(AA2=1;X2=1;Y2=1);"SK1(4)';IF(OR(AC2=1;AH2=1;AI2=1;AG2=0);"SK1(5)';IF(OR(BH2=1;AS2=1;AT2=1);"SK2(6)';IF(BI2=1;"SK2(7/8)';IF(AM2=1;"SK2(9)';IF(V2=1;"SK2(10)";"SK3(11)")))))

***Table S5: Parameter list for querying by the Excel syntax for the Jordanian-German project algorithm for the preclinical ((PETRA) SDC figure 4 (***[**28**](#_ENREF_28)***))***

| Query | Column |
| --- | --- |
| Walking + not pale + not burned + no sweat | BA + BC + BD + BE |
| Fatal injury | BB |
| Critical bleeding | AC |
| Clear airway | T and not intubated |
| Respiratory rate < 10 or > 30/ min | X and Y |
| Heart rate < 50 or > 110/ min | AI and AH |
| Disability |  |
| Unconscious | AO |
| Unresponsive | AO |
| T2 (yellow) |  |

MS Excel syntax for determining the triage categories for *PETRA (SDC* Figure 4 *(*[28](#_ENREF_28)*)* with variable reference to table 8. Queries from "fatal injury" to "RR under..." Not taken into account, as it has no influence on the triage category:

=IF(AND(BA2=1;BC2=0;BD2=0;BE2=0);"SK3(1)';IF(BB2=1;"EX(2)';IF(OR(X2=1;Y2=1);"SK1(5)';IF(OR(AI2=1;AH2=1);"SK1(6)';IF(AO2=0;"SK1(7)";"SK2(8)")))))

***Table S6: Parameter list for querying by the Excel syntax for PRIOR (***[**26**](#_ENREF_26)***)***

| Query | Column |
| --- | --- |
| Life-threatening bleeding | AC |
| Unconscious | AO |
| Unsafe airway | V |
| Breathing disorder | AA and AB |
| Apnoea | T |
| Respiratory rate clearly disturbed | Y and X |
| Circulatory disorder | AJ and AK (shock) |
| No radial pulse | AE or AG |
| Recap time > 2 s | AF |
| Heavy bleeding | AC |
| Unconsciousness | AN |
| Disoriented, somnolent | AN |
| Severe pain | AZ |
| Lying | BA |

MS Excel syntax for determining the triage categories for *PRIOR (*[26](#_ENREF_26)*), variable reference to table 9:*

=IF(AC2=1;"SK1(1)';IF(OR(AO2=0;V2=1);"SK1(2)';IF(OR(T2=0;AA2=1;AB2=1;Y2=1;X2=1);"SK1(3)';IF(OR(AJ2=1;AK2=1;AE2=1;AF2=1;AC2=1);"SK1(4)';IF(AN2=1;"SK1(5)';IF(AZ2=1;"SK1(6)';IF(BA2=0;"SK2(7)";"SK3(8)"))))))

***Table S7: Parameter list for querying by the Excel syntax for mSTaRT (***[16](#_ENREF_16)***)***

| Query | Column |
| --- | --- |
| Walkable | A |
| fatal injury | C |
| Breathing disorder | D |
| Respiratory rate | G |
| Radial pulse palpable | H |
| splashing bleeding | K |
| Follow instructions | L |

MS Excel syntax for determining the triage categories for *mSTaRT(*[16](#_ENREF_16)*), variable reference to table 10*

=IF(A=1;"SK3(1)';IF(C=1;"SK4(2)';IF(D=1;"SK1(3)';IF(G>30;"SK1(4)';IF(G<10;"SK1(4)';IF(K=1;"SK1(5)';IF(H=0;"SK1(6)';IF(L=0;"SK1(7)";"SK2(8)"))))))))

***Table S8: Levels of statistical significance for the comparison of the number of algorithm steps between the algorithms. ANOVA with post hoc Dunnet-T3 correction for inhomogeneity of variances:*** *White comparison independent of the triage category and color-coded according to the respective triage categories T1-T3****.***
